# Aiding Diagnosis and Classifying of Early Stage Osteonecrosis of the Femoral Head with Convolutional Neural Network Based on Magnetic Resonance Imaging

**DOI:** 10.1007/s43465-024-01272-7

**Published:** 2024-12-04

**Authors:** Chen Liang, Yingkai Ma, Xiang Li, Yong Qin, Minglei Li, Chuanxin Tong, Xiangning Xu, Jinping Yu, Ren Wang, Songcen Lv, Hao Luo

**Affiliations:** 1 Department of Orthopedics, Second Affiliated Hospital of Harbin Medical University, Harbin, Heilongjiang China; 2 Department of Control Science and Engineering, Harbin Institute of Technology, Harbin, 150001 China

**Keywords:** Osteonecrosis of the femoral head (ONFH), Diagnosis, ONFH classification, Convolutional neural network (CNN)

## Abstract

**Introduction:**

The Steinberg classification system is commonly used by orthopedic surgeons to stage the severity of patients with osteonecrosis of the femoral head (ONFH), and it includes mild, moderate, and severe grading of each stage based on the area of the femoral head affected. However, clinicians mostly grade approximately by visual assessment or not at all. To accurately distinguish the mild, moderate, or severe grade of early stage ONFH, we propose a convolutional neural network (CNN) based on magnetic resonance imaging (MRI) of the hip joint of patients to accurately grade and aid diagnosis of ONFH.

**Materials and Methods:**

T1-MRI images of patients diagnosed with early stage ONFH were collected. Three orthopedic surgeons selected 261 slices containing images of the femoral head and labeled each case with the femoral head necrosis classification. Our CNN model learned, trained, and segmented the regions of femoral head necrosis in all the data.

**Results:**

The accuracy of the proposed CNN for femoral head segmentation is 97.73%, sensitivity is 91.17%, specificity is 99.40%, and positive predictive value is 96.98%. The diagnostic accuracy of the overall framework is 90.80%.

**Conclusions:**

Our proposed CNN model can effectively segment the region where the femoral head is in MRI and can identify the region of early stage femoral head necrosis for the purpose of aiding diagnosis.

## Introduction

Osteonecrosis of the femoral head (ONFH) is a common disease resulting in localized death of osteocytes and bone marrow components due to venous stasis or impaired arterial blood supply to the femoral head [1]. As the disease progresses, structural deterioration and collapse of the femoral head can lead to hip pain and dysfunction. In the United States, the cumulative number of patients with ONFH is approximately 300,000–600,000 [2]. Each year, 12,000–24,000 new cases of osteonecrosis are diagnosed in Japan and the average annual number of new cases is thought to be 14,103 in South Korea [3]. Epidemiological surveys in China have shown the cumulative number of ONFH patients is estimated at 8.12 million in people over 15 years of age; glucocorticoids, alcohol, smoking, obesity, certain occupations, and diabetes are high-risk factors for ONFH [4, 5]. Among ONFH patients, prevalence is higher in Chinese men than women and higher in northern residents than in southern [6].

Meanwhile, studies on the management of early to intermediate-stage ONFH have proposed a variety of treatments, among which surgery can be effective in improving symptoms and slowing or stopping the progression of ONFH [7]. In particular, osteotomies [8] combining core decompression (CD) with other techniques [9], such as bone marrow-derived cell therapies (BMCTs) [10], are the most commonly reported surgical procedures for early to moderate ONFH. This is despite the fact that most patients with early to moderate ONFH in the available studies have reported positive responses to surgical treatment, such as overall pain reduction and improvements in movement and gait. It is still inevitable that some patients will progress and eventually require total hip arthroplasty (THA) to resolve ONFH.

This shows that with such a large number of patients, the diagnosis of ONFH is particularly important, especially for early stage ONFH. In addition, there is evidence that a longer duration of symptoms before treatment is one of the negative prognostic factors after treatment for ONFH [11]. Therefore, early detection, early treatment, and early prevention of ONFH progression are the only ways to reduce the medical burden on society and improve the quality of life of patients. Magnetic resonance imaging (MRI) is very sensitive to early changes in ONFH and has become one of the most important tools for clinicians to screen for ONFH [12, 13]. The Steinberg classification [14] is commonly used by orthopedic surgeons to assess the severity of ONFH patients, and more detailed grading of early ONFH has been performed based on the abnormal area of the femoral head on MRI.

However, in actual clinical practice, orthopedic surgeons or radiologists do not fully follow the Steinberg classification to diagnose the severity of ONFH patients. First, due to the excessive pressure of clinical diagnosis and the huge number of patients, it is impossible to achieve an accurate classification diagnosis; second, the ratio of the area of femoral head involvement to the total femoral head area reflected by hip MRI in early ONFH patients is not accurately labeled, and doctors can only roughly estimate it, resulting in the severity of early ONFH patients being highly associated with the subjectivity of doctors, and therefore may be misjudged. At the same time, pixel-level segmentation of the femoral head region and the necrotic region is a time-consuming and labor-intensive task. Therefore, there is an urgent need for complementary diagnostic methods that can accurately detect the presence and quantify the severity of early ONFH.

Convolutional neural network (CNN) has been validated in medical image recognition because of its powerful image processing ability to rapidly compute and recognize the content of medical image data, and it has shown great effectiveness in glaucoma detection [15], detection of diseased tissue in lung CT of COVID-19 patients [16], and even in predicting anticancer drug sensitivity [17]. Thus, CNN can also identify the presence of lesions in hip MRI of early ONFH patients, which can help clinicians make a diagnosis. With the help of fully automated diagnosis and grading CNN, the diagnostic efficiency can be effectively improved, and no fatigue problem will occur in long-term diagnosis.

The purpose of our study is to develop a novel CNN model to aid in the diagnosis and classification of early stage ONFH. The proposed model is evaluated and compared with existing CNN models, such as LinkNet and U-Net.

## Materials and Methods

**Fig. 1:**
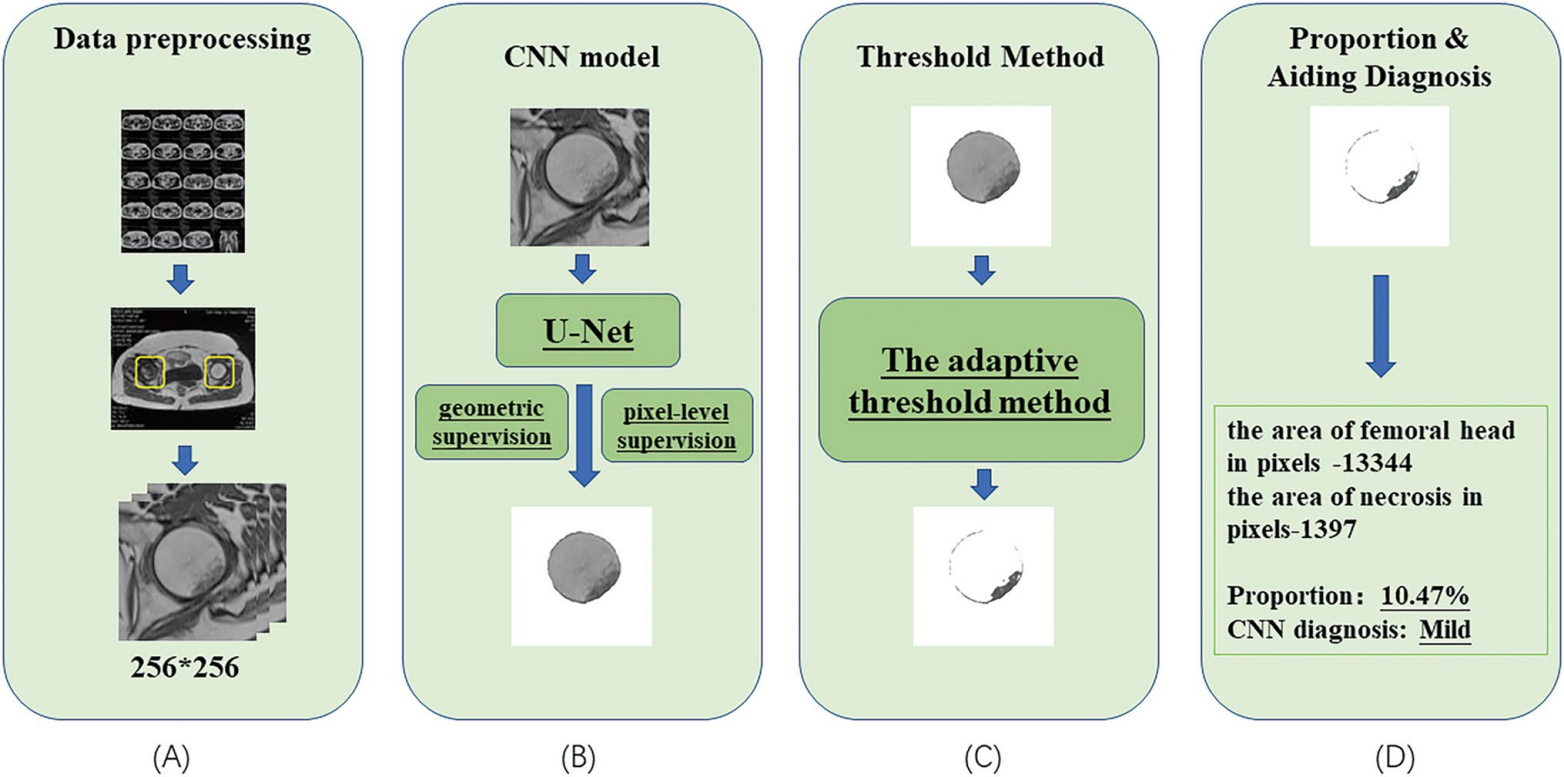
Network architecture of our proposed CNN model consisted of four main components: **A** data preprocessing: the hip axial MRI-T1 phase data of the selected patients were individually segmented into the individual femoral head images as the smallest input data unit; **B** CNN identified and segmented the femoral head regions. Based on the U-Net model, we added geometric supervision and pixel-level supervision to the CNN model, both of which were independent paths to enhance the accuracy of femoral head region segmentation; **C** adaptive thresholding method could grab necrotic regions, which could automatically select the best threshold to distinguish normal bone tissue from necrotic regions in the femoral head region; **D** proportion and aiding diagnosis based on the proportion between the area of necrotic regions and all area of the femoral head region at the pixel level, and further outcomes included the severity grading of the disease and the auxiliary diagnostic suggestion

This retrospective study provides level III evidence. MRI data of patients were collected from the Department of Minimally Invasive Surgery and Sports Medicine of The Second Affiliated Hospital of Harbin Medical University, and the MRI data of 261 femoral heads were finally obtained after acquiring an institutionalreview board (IRB) approval. Patient selection criteria: (1) inclusion criteria: patients were diagnosed with early stage ONFH according to Steinberg classification and Chinese Guideline for Diagnostic and Treatment of Osteonecrosis of the Femoral Head[18]; no abnormal femoral head signs were seen on Hip X-rays; the interval between MRI and X-ray was 1 week or less. (2) Exclusion criteria: patients’ age was less than 18 years; the interval between MRI and X-ray was longer than 1 week; patients were diagnosed with Osteoarthritis (OA). The preprocessing of the collected data is shown in Fig. 1A. Three orthopedic surgeons screened the data, and each layer of the patient’s MRI-T1 images was separated into one data, after which each hip image was also separated to become the final data set, with a total of 261 axial MRI-T1 pieces of femoral head image data. A chief orthopedic surgeon examined the data set to determine the accuracy, after which all data were normalized and each case was cropped to 256 × 256 images to make the data set consistent in image size. The training of the CNN model was then performed, and in this task, 80% of the images were randomly selected as the training set and the remaining 20% as the test set.

When our image entered the CNN model, its pixel information was converted into a matrix of pixel numbers. Instead of recognizing the image directly, the artificial intelligence learned and trained by extracting features from the digital information of the image and then made a judgment. Thus, the CNN extracted the digital information of the key regions of the image, and since these key regions determined the classification of the disease, the CNN could compare the pre-trained criteria with the extracted information and finally classify them for clinically assisted diagnosis. Overall, the learning process of our model was the result of the model learning on 80% of the data (the training set mentioned in Materials and Methods), our input data for the training set were all MRI images of ONFH patients, and the training process was the model testing on the unlearned training set (20% of the data) and detecting the results of the training (Table 1). The model then segmented the necrotic regions in the femoral head images using an adaptive thresholding method and calculated their area percentage.

**Table 1:** Performance of our proposed CNN model

CNN model	Accuracy	Sensitivity	Specificity	PPV
LinkNet	96.68%	88.67%	98.75%	94.09%
U-Net	97.42%	89.19%	99.12%	95.77%
Ours	97.73%	91.17%	99.40%	96.98%

For this task, we evaluated our CNN model using the following evaluation metrics, including accuracy, sensitivity, specificity, and positive predictive value (PPV).

Our proposed task framework consists of two main parts, the first part was to accurately segment the region of the femoral head with a CNN model based on U-Net [19], and the second part was to extract the necrotic tissue in the femoral head region using an adaptive threshold method. First, the U-Net was a classical CNN model that was proposed in 2015, and it successfully demonstrated its effectiveness in segmenting target images even when training a small number of input images using data augmentation. In addition, the U-Net ran very fast and took less than one second to segment a image [19]. Based on the U-Net, our CNN model added two additional supervision modes, namely, geometric supervision and pixel-level supervision to the output results, and each of the two supervision modes was an independent path as a constraint to enhance the segmentation accuracy of the femoral head region. Second, after the femoral head region segmentation was completed, the output results entered the second part of the network structure. The adaptive threshold method would automatically calculate the optimal threshold to segment the necrotic part based on the distribution characteristics of the normal bone and necrotic part of the femoral head region and finally calculated the proportion of the necrotic part and the image with digital results. The flowchart of our proposed task is shown in Fig. 1.

## Results

For our proposed femoral head segmentation task, four evaluation metrics were used to evaluate our CNN model, including accuracy, sensitivity, specificity and positive predictive value (PPV). The performance of our proposed CNN model for femoral head segmentation and threshold method shown in Table 1, which accuracy was 97.73%, sensitivity was 91.17%, specificity was 99.40%, positive predictive value was 96.98%. Compared with the U-Net and LinkNet models, ours got superior performance in each evaluation metric by analyzing all data.

**Fig. 2:**
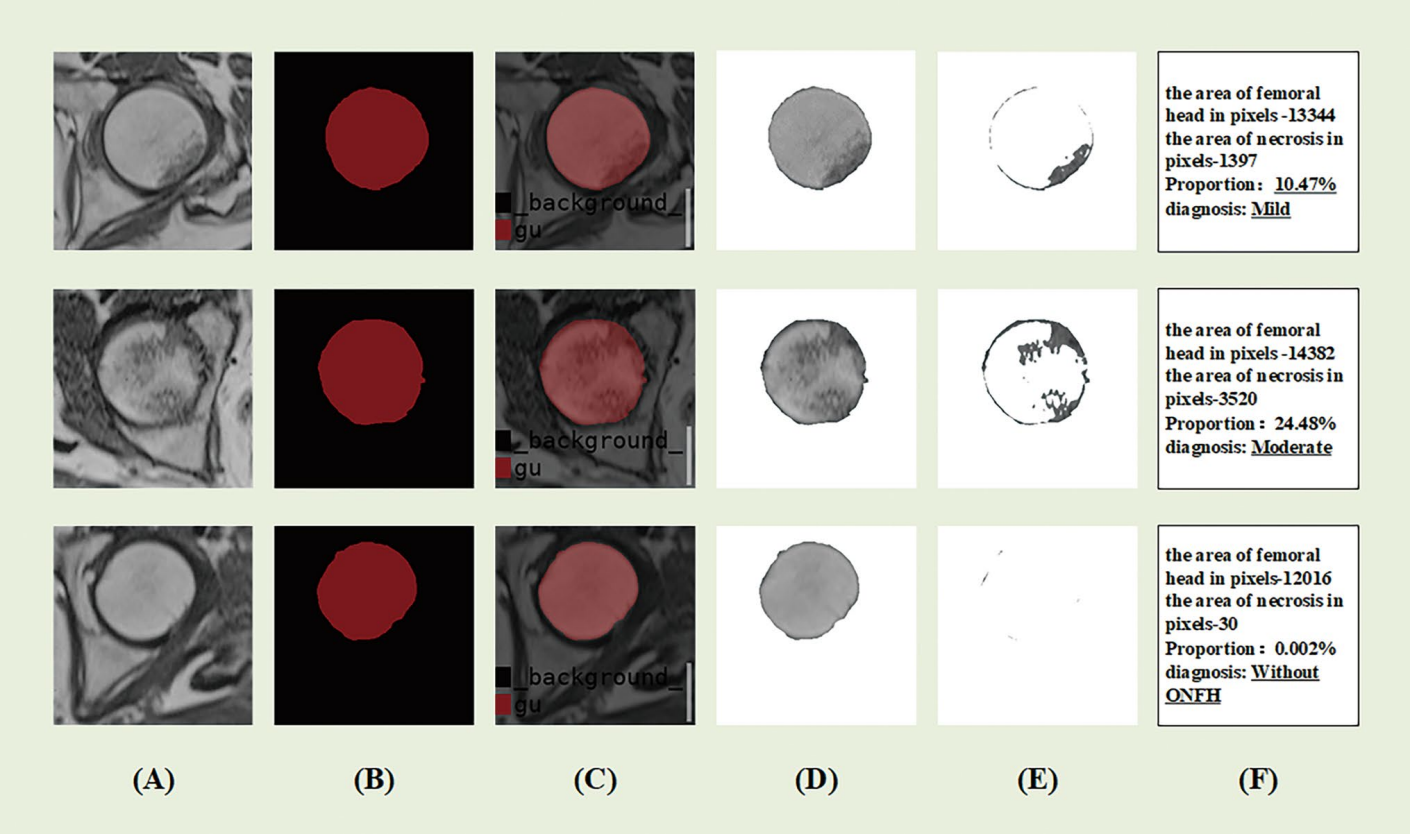
Procedure of outcomes analyzed by our model. **A** Input data of femoral head images. **B** Segmentation of the femoral head. **C** Visualization of segmentation results. **D** Extracted images of the femoral head region. **E** Result of the adaptive threshold method. **F** Finished output and diagnostic recommendations

The analysis process of the input data in our model is shown in Fig. 2. First, the MRI images of the femoral head after data preprocessing were entered into the CNN model with the same size after normalization; after that, the black part of the segmentation result was the background part recognized by the model, such as other bone tissues and muscle tissues in the non-femoral head region, and the red part was the femoral head region; after that, the femoral head region image was extracted and entered into the second half of the model, and the adaptive thresholding method was used to distinguish the normal bone tissue from the necrotic part; finally, according to the size of necrotic part at the pixel level, further calculations were performed and the auxiliary diagnostic results were output according to Steinberg classification (Mild: Proportion < 15%; Moderate: 15% ≤ Proportion ≤ 30%; Severe: Proportion > 30%). Meanwhile, the last line in Fig. 2 shown that there was no ONFH sign in the input femoral head image, and we set the output result to “Without ONFH” when the final calculation result was < 0.1%, and the visualization results were output at the same time to further aiding clinicians in diagnosis.

## Discussion

In this paper, we proposed a new CNN model based on U-Net, which first accurately segmented the femoral head in the hip MRI of patients with early stage ONFH, second identified the necrotic part in the femoral head region, and accurately calculated the percentage of necrotic part, and finally outputs the ONFH images identified by the CNN model and the severity grading of the disease according to Steinberg classification. Although our CNN model added supervision and adaptive threshold methods to U-Net, which increased the computational elements in the whole network structure and the computing time, the same recognition task took longer time than the U-Net, but this was still faster than that of manual labeling, and there is no error problem due to fatigue. Different from the past, our CNN model simplified the calculation of ONFH volume. It is more convenient and accurate in ONFH severity grading than a surgeon in processing large numbers of images. Our CNN model can assist medical centers with emergency mass imaging diagnosis and staging, or assist medical workers in diagnosis, treatment, or teaching in local medical facilities where there is no specialist assistance.

Regarding the artificial intelligence-aided diagnosis of ONFH, researchers have carried out studies, and well performance of the assisted diagnosis has been achieved for different data types. Li et al. [20] set up an ROI (region-of-interest) module to manually label the femoral head region before identification for hip radiographs to improve the diagnostic accuracy, and the results showed that the model was effective and significantly improved the diagnostic time of orthopedic surgeons. Zhu et al. [21] used hip computed tomography (CT) as training data and trained and identified femoral head CT sections of people aged 45–60 years with a CNN model based on multi-source neural networks like LeNet and U-Net, and then achieved the purpose of segmenting the femoral head region. They divided the data into three types of training, a circular section of the femoral head in the upper part of CT; a section of the femoral head and femoral neck in the middle part of CT; and an irregular circular section of the femoral head in the lower part of CT, and extracted 3D femoral head features. The CNN model segmented the femoral head with high accuracy, and the usability of the model was enhanced by adding a data dimensionality reduction structure to the network. Chee et al. [22] trained a deep CNN model based on ResNet with MRI images of the femoral head from dual centers, and the sensitivity of diagnosing ONFH was no less than that of inexperienced and experienced radiologists. In addition, in the analysis of the CNN model femoral head collapse or not, the diagnostic sensitivity was higher for precollapse femoral head patients than for less experienced radiologists. Wang et al. [23] also used U-Net to train the coronal position of the hip MRI, and the network structure was enhanced with point rendering to enhance the segmentation accuracy aimed at the femoral head. This study excluded patients with advanced ONFH with femoral head collapse (ARCO IIIB) and hip osteoarthritis (ARCO IV) and achieved good results for early ONFH identification. Nevertheless, compared to our proposed CNN model, the output of our network can directly extract the necrotic portion of the femoral head, the visualization of the results is more straightforward, the reliability of the results is higher in a clinical setting, its ability to aid diagnosis is simpler and more straightforward when used in teaching for inexperienced clinicians or in areas where medical resources are relatively scarce, and our model has higher specificity in the femoral head segmentation task.

However, in the current practical clinical environment, orthopedic surgeons or radiologists cannot accurately diagnose the severity of ONFH patients according to Steinberg classification.

First of all, the number of patients is huge, according to statistics, the number of patients with ONFH in China is as high as 8.12 million. The radiologist needs to make a report on the patient’s imaging information quickly, and the orthopedic surgeon needs to make a final diagnosis on the patient as soon as possible based on the patient’s imaging information and report after a complete evaluation. Thus, the pressure of clinical diagnosis is too heavy, and the severity of the condition of patients with ONFH cannot be realized by rapid and accurate grading of diagnosis, patients can only be informed with “yes or no”, and the specific grading of diagnosis cannot be taken into account; for the sake of efficiency of diagnosis in a real clinical environment, most of the orthopedic surgeons and doctors choose to ignore the precise grading of diagnosis.

Second, with the development of online media, a huge amount of ONFH-related content has been spread on the Internet, so patients’ attitudes have also changed. In the past, when patients experienced hip pain and discomfort, their first choice for symptomatic treatment was medication, mainly analgesics and Chinese medicine creams; now, when patients experience the same situation again, their first reaction is to search for content similar to their symptoms on the online platforms of their mobile phones. At the same time, most orthopedic surgeons and radiologists also publish ONFH popularization content and teaching content of professional medical knowledge on the Internet under strict control. As a result, patients will get a rough idea about ONFH by reading a lot of relevant content. As a result, patients’ demands for grading their conditions are gradually increasing, which further increases the diagnostic pressure on orthopedic surgeons and radiologists. In addition, some patients are skeptical and reject doctors’ vague diagnoses, which inevitably aggravates the already tense doctor–patient relationship and leads to more conflicts.

In addition, the ratio of the femoral head lesion area to the total femoral head area as reflected by hip MRI in patients with ONFH cannot be accurately labeled at present, and clinicians can only roughly estimate it, resulting in the severity of patients with early stage ONFH being highly correlated with clinician subjectivity, which may lead to misclassification. Undeniably, pixel-level segmentation of the femoral head area and necrotic area is an even more time-consuming and laborious task.

Therefore, our proposed model can be fully utilized in the clinical environment, which can not only quickly and accurately segment the necrotic regions of the femoral head from the pixel level to assist clinicians in diagnosis, but also can accurately display the proportion of necrotic regions, which makes hierarchical diagnosis possible, and further reduce the diagnostic pressure of clinicians, and also to some extent can make patients have more confidence in their doctors to alleviate the tense doctor–patient relationship. When our proposed model learns and trains more clinical data, it can also become a teaching tool for resident doctors and achieve the purpose of medical professional education.

Nonetheless, our study is not without limitations. First, we only targeted axial MRI of the hip, in which coronal and sagittal data were not combined. Since MRI data are three-dimensional, not fully utilizing the patient’s data may have some impact on the final assisted diagnosis results. In addition, the training data set in this study was relatively less and there was no suitable data set that could be used as an external validation data set for the model to further validate the aiding diagnostic accuracy of our model, but our test results demonstrated that our model achieved acceptable aiding diagnostic accuracy. In the long view, collecting more patient data with corresponding clinical information and enhancing external validation to strengthen the generalization capability of the model are expected to further improve the model performance.

## Conclusion

In this paper, we apply a customized CNN model for the ONFH of segmentation and aiding diagnosis of ONFH grading. Its performance is achieved on both ONFH diagnosis, and its aiding ONFH grading could help Orthopedists make a therapeutic tactic for ONFH patients. Compared to the variants of U-Net, our proposed CNN model achieves better performance. Although our model has demonstrated convincing performance, it still needs to be run in a real clinical environment and adapted to the needs of clinicians to achieve its purpose of assisting clinicians.

## Data Availability

The data sets used and analyzed during the current study available from the corresponding author on reasonable request.
